# Regulation of Ov2 by virus encoded microRNAs

**DOI:** 10.1007/s11259-019-09749-9

**Published:** 2019-03-19

**Authors:** Katie Nightingale, Inga Dry, John Hopkins, Robert Dalziel

**Affiliations:** 1 The Roslin Institute & R(D)SVS, University of Edinburgh, Edinburgh, Midlothian, EH25 9RG UK; 2 Present Address: Cambridge Institute of Medical Research, Hills Road, Cambridge, CB2 0XY UK

**Keywords:** Ovine herpesvirus-2, Virus encoded miRNAs, Ov2

## Abstract

Herpesviruses encode miRNAs that target both virus and host genes; however their role in herpesvirus biology is still poorly understood. We previously identified thirty five miRNAs encoded by OvHV-2; the causative agent of malignant catarrhal fever (MCF) and are investigating the role of these miRNAs in regulating expression of OvHV-2 genes that play important roles in virus biology. Analysis, using RNAHybrid predicted that two OvHV-2 encoded miRNAs, ovhv2-miR-17-10 and ovhv2-miR-61-1, target transcripts coding for the OvHV-2 bZIP protein Ov2. In other herpesvirus bZIP proteins are known to play important roles in lytic virus replication. Here we show by Flow cytometry and western blotting that ovhv2-miR-17-10 and ovhv2-miR-61-1, reduce the expression of Ov2 protein. The predicted target sites for both miRNAs within the Ov2 gene were disrupted whilst retaining the Ov2 coding sequence. Mutation of the ovhv2-miR-61-1 target sequence restored Ov2 protein expression levels to control levels confirming the identity of its target site. However, it was not possible to determine the binding site of ovhv2-miR-17-10 possibly due to potential G:U pairing introduced during the mutation process. The targeting of Ov2 by two virus-encoded miRNAs suggests an important regulatory role for Ov2 in OvHV-2 replication or reactivation.

## Introduction

Malignant catarrhal fever (MCF) is a usually fatal disease of cattle, deer, bison and other ruminants caused by viruses in the genus *Macavirus* of the subfamily *Gammaherpesvirinae* (McGeoch et al. 2006). MCF is characterised by sudden onset of fever followed by lymphadenopathy, leucocytosis, severe congestion and necrosis and erosion of the oral, conjunctival and nasal muscosæ (Russell et al. 2009). The major causative agents of MCF are ovine herpesvirus 2 (OvHV-2) and alcelaphine herpesvirus 1 (AlHV-1), which cause the sheep associated (SA-MCF) and the wildebeest associated (WA-MCF) forms of the disease, respectively. In the case of both the SA and WA- forms of MCF, reactivation of the virus from latency results in the production of infectious virions that can be transmitted to susceptible species, such as cattle, bison, or deer found in close proximity to asymptomatically shedding carrier animals.

Reactivation of gammaherpesviruses from their latent state requires the expression of viral transactivators, such as the Replication and Transcriptional Activator protein (RTA), that promote virus replication by triggering expression of a cascade of proteins that are required for viral genomic replication and particle formation (Damania et al. 2004). The Ov2 gene of OvHv-2 encodes a protein with a basic leucine zipper (bZIP) domain. Herpesvirus proteins with bZIP domains include BZLF1 of Epstein Barr Virus (EBV) (Murata 2014), and the K8 protein of Kaposi’s sarcoma-associated herpesvirus (KSHV) (Lefort and Flamand 2009). BZLF and K8 play important roles in the lifecycles of EBV and KSHV by modulating the activity of RTA (Liao et al. 2003; Murata 2014).

MicroRNAs (miRNAs) are short, non-coding RNAs that regulate gene expression at a post-transcriptional level. miRNA-mediated regulation of expression is by binding of the seed sequence (usually nucleotides 2 to 8) to complementary sequences in the target mRNA and directing these targets for degradation or translational silencing, depending on the degree of complementarity observed (Bartel 2009). MicroRNAs have been identified in the genomes of many virus families, though, it is the *Herpesviridae*, with over 300 viral microRNAs identified so far amongst its members, that seem to have evolved to make the greatest use of miRNAs as a mechanism to effectively regulate cellular and virus gene expression (Grundhoff and Sullivan 2011). Independent research into the targets of the miRNAs encoded by herpesviruses have identified in the genomes of viruses classified within all herpesvirus sub-families, the presence of miRNAs which target the viral transactivators (Bellare and Ganem 2009; Grey et al. 2007; Umbach et al. 2008). This is indicative of a familial level mechanism regulating the balance between herpesvirus lytic and latent replication cycles.

Previously our group has shown that OvHV-2 encodes for at least thirty-five miRNAs, and that the targets of these miRNAs include transcripts encoding the OvHV-2 RTA (encoded by the ORF50 transcript) (reactivation) and ORF73 (maintenance of latency) (Nightingale et al. 2014; Riaz et al. 2014). Further analysis, using RNAHybrid (Rehmsmeier et al. 2004) predicted that two further OvHV-2 encoded miRNAs, ovhv2-miR-17-10 and ovhv2-miR-61-1, would target transcripts coding for the OvHV-2 protein Ov2.

In this paper, we show that two of the thirty-five identified OvHV-2-encoded miRNAs, ovhv2-miR-17-10 and ovhv2-miR-61-1, target the virus-encoded protein Ov2. Furthermore, the fact that two virus-encoded miRNAs target Ov2 is suggestive of an important regulatory role for Ov2 in the process of OvHV-2 reactivation.

## Methods

### Cell culture

BJ1035 cells, an immortalized bovine T cell line from an animal naturally-infected with OvHV-2 [3], was grown in suspension culture in Iscove’s Modified Dulbecco’s Medium (Invitrogen, UK) supplemented with 10% (*v*/v) foetal calf serum (FCS, Sera Laboratories International, UK), 1% (v/v) penicillin-streptomycin (Invitrogen), 20 U/ml interleukin 2 (Novartis Pharmaceuticals UK) and incubated at 37 °C, 5% CO_2_. Human embryonic kidney cells (HEK-293 T) were cultured in Dulbecco’s Modified Eagle Medium (Invitrogen) supplemented with 10% (v/v) FCS and 1% (v/v) penicillin-streptomycin-glutamine (Invitrogen) and incubated at 37 °C, 5% CO_2_.

### Target identification

The nucleotide sequence of Ov2 was derived from the OvHv-2 genome (Genbank accession: AY839756.1). Sequences and position of the OvHV-2-encoded miRNA in the OvHV-2 genome have been described previously (Nightingale et al. 2014). RNAHybrid (Rehmsmeier et al. 2004) was used to predict OvHV-2-encoded miRNA target sites within the coding region of Ov2. No G:U pairing in the seed sequence was allowed, and a helix constraint of nucleotides 2 to 8 was used.

### Primer design and cloning

The OvHV-2 OV2 gene sequence amplified by PCR from DNA extracted from OvHV-2 positive BJ1035 cells (Schock et al. 1998) using the DNA Blood and Tissue kit (Qiagen, UK). The reaction contained 1 unit HotStarTaq *Plus* DNA polymerase (Qiagen), 50 ng BJ1035 DNA, 200 μM dNTPS and 8 pmols of each primer in a final reaction volume of 20 μl. 

The cycling conditions used for amplification were an initial denaturation step of 5 min at 95 °C, followed by 30 cycles of 95 °C for 30 s, 58 °C for 1 min and 72 °C for 1 min, with a final extension of 72 °C for 7 min.

Untagged Ov2 was cloned into pcDNA3.1(+) using the restriction enzymes *BamH1* and *EcoR1*. Ov2 was cloned in frame with EGFP, in the vector pEGFPN1, using the restriction enzymes *Bgl II* and *EcoR1*. Sequencing by GATC (Cologne, Germany) confirmed the absence of any additional mutations in the Ov2 sequence.

### Site directed mutagenesis

Site-directed mutagenesis PCR was carried out using the QuikChange Lightning Site-Directed Mutagenesis Kit (Agilent Technologies, UK) according to the manufacturer’s protocol. Mutagenesis primers (Table 1) were designed using the QuikChange Primer Design Program (www.agilent.com/genomics/qcpd). All mutations were confirmed by sequencing by GATC.

**Table 1:** Oligonucleotide primers used in experimental design

Primer name	Primer Sequence	Use
Ov2 ovhv2-miR-17-10 binding site mutagenesis Forward	GCTCCTGTAACCTCTTCTTAAAATTGCGTGATGCCCTTCTATTTGTGCTCCTGC	mutation of Ov2 ovhv2-miR-17-10 binding site
Ov2 ovhv2-miR-17-10 binding site mutagenesis Reverse	GCAGGAGCACAAATAGAAGGGCATCACGCAATTTTAAGAAGAGGTTACAGGAGC	mutation of Ov2 ovhv2-miR-17-10 binding site
Ov2 ovhv2-miR-61-1 binding site mutagenesis Forward	CATCCACTCCCACCTACCTGAGGTGACCATAAATGTTGTTCGGAGTTGTAAAAATCATTTGT	mutation of Ov2 ovhv2-miR-61-1 binding site
Ov2 ovhv2-miR-61-1 binding site mutagenesis Reverse	ACAAATGATTTTTACAACTCCGAACAACATTTATGGTCACCTCAGGTAGGTGGGAGTGGATG	mutation of Ov2 ovhv2-miR-61-1 binding site
Ov2 Fwd	GGATCCACCATGTCCGATAATAAAAAGC	For cloning into pcDNA3.1+
Ov2 Rev	GAATTCACGCGTCTACAAGCTGTGTAACATATTCAACTCC	For cloning into pcDNA3.1+
Ov2 BglII fwd	TCAGATCTCATGTCCGATAATAAAAAGC	For cloning into pEGFPN1
Ov2 Ecor1 Ov2 rev	GAATTCGCAAGCTGTGTAACATATTCAACTCC	For cloning into pEGFPN1

### Flow cytometry

Ten ng plasmid DNA and 100 nM miRNA mimic (Nightingale et al. 2014) or 100 nM AllStars Negative Control siRNA (both Qiagen) were transfected into HEK-293 T seeded at 3 × 10^4^ cells per well of a 96 well dish, using Lipofectamine 2000 (Invitrogen). Cells were detached using trypsin 48 h after transfection, washed and resuspended in 1% (*w*/*v*) PBS/BSA. Samples were analysed using an LSRFortessa High Throughput Sampler (BD Biosciences). Forward scatter and side scatter were used to gate live cells. A mock transfected cell population was used as a negative control. Data were analysed using FlowJo v10.0.6. Median fluorescence intensity (MFI) was used as a measure of expression. 7500 GFP-positive cells were counted with *n* = 14 for each condition.

### Western blotting

One hundred ng plasmid DNA and 100 nM miRNA mimic (Nightingale et al. 2014) or 100 nM AllStars Negative Control siRNA (both Qiagen) were transfected using Lipofectamine 2000, into 2 × 10^5^ HEK-293 T cells per well of a 12 well dish. Samples were harvested in Laemmli Buffer (Bio-Rad, UK) and boiled for 5 min at 95 °C before separation by SDS-PAGE using 4–20% Mini-PROTEAN® TGX™ Gels (Bio-Rad). Membranes were probed with either rabbit anti-Ov2 antibody (synthesised using peptides RRSTNRRASRNFKKRLQEH (amino acids 26–45, accession # AY839756.1) and EKKKEEIRRLQYWLSTHNC, (amino acids 67–85 accession # AY839756.1) peptides by Dundee Cell Products, UK) or a mouse anti-beta Actin (mAbcam 8226) and appropriate fluorescent secondary antibodies (LI-COR). Primary (1:1000) and Secondary antibodies (1:10000) were diluted in PBS-T (PBS + 0.1% Tween 20 *v*/v). Protein bands were visualised using an Odyssey CLx (LI-COR) and quantified using Image Studio Lite version 3.1 (LI-COR). For each sample, the levels of Ov2 were quantified and normalised to the level of actin. Test ovhv2-miRNA samples were then compared to the negative control siRNA. A total of three independent western blots were performed with duplicates for each condition.

### Statistical analysis

Statistical analysis was performed using Minitab 17 software. Differences between groups were analysed using a general linear model followed by Tukey’s post-hoc test. *P*-values represent results from the post-hoc test.

### Illustrations

Illustrations were generated using GraphPad Prism.

## Results

### Control of Ov2 expression by OvHV-2 encoded miRNAs

**Fig. 1:**
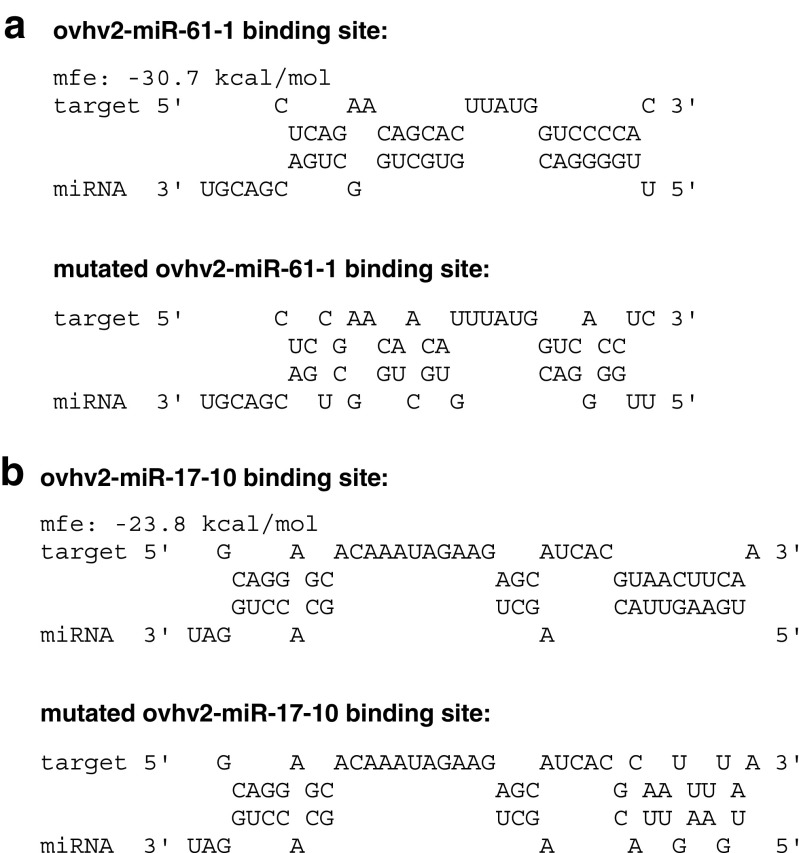
**RNAHybrid analysis showing predicted binding of ovhv2-miR- 61-1 and ovhv2-miR- 17-10 to Ov2 target sequence**. RNAHybrid analysis showing predicted binding, with minimum free energys (mfe) of ovhv2-miR- 61-1(**a**) and ovhv2-miR- 17-10 (**b**) to the Ov2 target sequence. The RNAHybrid analysis showing predicted binding ovhv2-miR-61-1 (**a**) and ovhv2-miR-17-10 (**b**) to a mutated Ov2 target sequence is shown underneath. Constraints on miRNA binding included perfect complementarity between nucleotides 2 and 8 and no G:U pairing within this region

The capacity of two OvHV-2-encoded miRNAs (ovhv2-miR-17-10 (minimum free energy, mfe = −23.8 kcal/mol) and ovhv2-miR-61-1 (mfe = −30.7 kcal/mol), Fig. 1), to affect Ov2 expression was investigated .

**Fig. 2:**
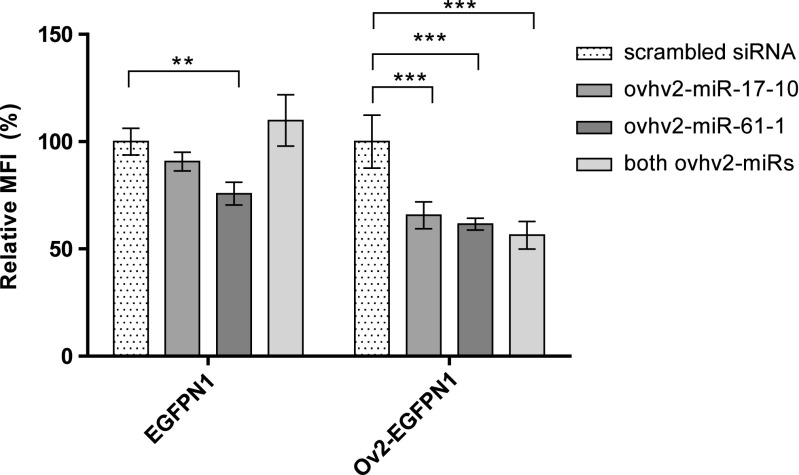
**Confirmation of Ov2 targeting by miRNAs using Flow cytometry.** 293 T cells were co-transfected with either an EGFPN1 or an Ov2-EGFPN1 construct and ovhv2-miR-17-10, ovhv2-miR-61-1, a combination of both ovhv2-miRs or a scrambled siRNA. The MFI for each sample was determined 48 h later by flow cytometry and test ovhv2-miRNAs were compared to a scrambled siRNA control. ** = *p* ≤ 0.01, *** = *p* ≤ 0.0001. Error bars show ± SEM. MFI = Mean Fluorescence Intensity

HEK-293 T cells were transfected with Ov2-EGFPN1 and either ovhv2-miR-17-10 mimic, ovhv2-miR-61-1 mimic, a mixture of both or a negative control siRNA. Forty-eight hours post transfection GFP expression was analysed by flow cytometry as described above. Overall, the percentage of GFP positive cells was found to remain consistent between replicates and conditions (data not shown). To control for off-target effects, the ability of the OvHV-2 miRNA mimics to reduce expression of GFP alone was also examined. No significant changes in the MFI were observed when ovhv2-miR-17-10 or a combination of both ovhv2-miRs were co-transfected with an EGFPN1 construct compared to a negative control siRNA (Fig. 2). A decrease of 25% (*p* ≤ 0.01) in the MFI was observed when ovhv2-miR-61-1 was co-transfected with an EGFPN1 construct compared to the negative control siRNA (Fig. 2). Significant decreases (*p* ≤ 0.0001 for all) of 35%, 39% and 44% in the MFI were observed when ovhv2-miR-17-10, ovhv2-miR-61-1 or a combination of both ovhv2-miRs respectively were co-transfected with an Ov2- EGFPN1 construct compared to a negative control siRNA (Fig. 2).

As there were potential off target effects of the ovhv2-miR-61-1 on EGFP we investigated the effects of the OvHv-2 miRNA mimics to regulate expression of an untagged version of Ov2, by western blot analysis using an Ov2 specific antibody. Using this approach, the ability of either ovhv2-miR-17-10 or the ovhv2-miR-61-1 to reduce expression of Ov2 transcripts that had either a disrupted ovhv2-miR-17-10 or ovhv2-miR-61-1 binding site was also examined. In each case the mutagenesis was designed to mutate the binding site, without impacting on the coding sequence of Ov2 (Fig. 1).

**Fig. 3:**
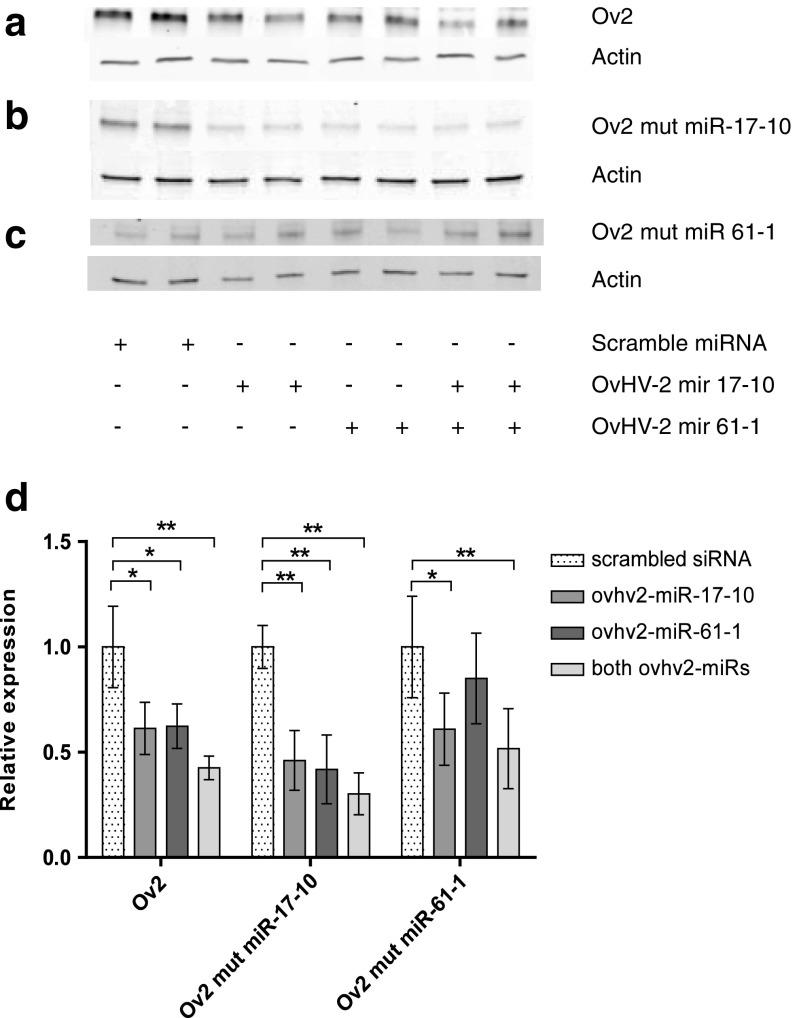
**Analysis of miRNAs targeting Ov2 by western blotting.** HEK-293 T were transfected with the expression construct Ov2-pcDNA3.1+ and the appropriate miRNA mimic or a scramble siRNA. Representative western blots of protein extracts from cells transfected with (**a**) Ov2-pcDNA3.1+, (**b**) mutated Ov2-mut -mIR-17-10 or (**c**) Ov2-mut-miR-61-1 with ovhv2-mIR-17-10, ovhv2-miR-61-1 or a combination of both are shown. (**d**) Ov2 was detected using an anti-Ov2 antisera and quantified using the LI-COR system. Expression of Ov2 was normalised to expression of actin for each sample. Levels of expression in the presence of test ovhv2-miRs were compared to a scrambled siRNA. * = *p* ≤ 0.05, ** = *p* ≤ 0.01, *** = *p* ≤ 0.0001

When either ovhv2-miR-17-10 or ovhv2-miR-61-1 were co-transfected with an Ov2- pcDNA3.1(+) construct significant reductions of approximately 40% (*p* ≤ 0.05 for both ovhv2-miRNAs) were observed compared to the negative control siRNA. A reduction of approximately 60% was observed compared to the negative control siRNA when both ovhv2-miR-17-10 and ovhv2-miR-61-1 were co-transfected with the Ov2- pcDNA3.1(+) construct (*p* ≤ 0.01). Restoration of expression of Ov2 was observed when ovhv2-miR-61-1 was co-transfected with Ov2-mut-miR-61-1 (*p* ≤ 0.58), though this construct remained sensitive to the presence of ovhv2-miR-17-10 (*p* ≤ 0.05; Fig. 3). However, restoration of Ov2 expression was not observed when Ov2-mut-miR-17-10 was expressed in the presence of either ovhv2-miR-17-10 or ovhv2-miR-61-1 (*p* ≤ 0.01; Fig. 3).

## Discussion

Evidence is accumulating from a number of herpesvirus, including HCMV and KSHV, that virally-encoded miRNAs may function like a rheostat, in fine-tuning the mechanism of reactivation by targeting viral transactivators (Grey et al. 2007; Bellare and Ganem 2009; Riaz et al. 2014). A similar role for miRNAs, in increasing stringency to biological pathways, has also been postulated for cellular miRNAs (Ebert and Sharp 2012). In this study, ovhv2-miR-17-10 and ovhv2-miR-61-1 have been shown to target Ov2. Mutation of the target seed sequence of ovhv2-miR-61-1 restored Ov2 expression levels to that of the control confirming that the target site of ovhv2-miR-61-1, within the coding sequence of the Ov2 mRNA, had been successfully identified. However, it was not possible to determine the functional binding site of ovhv2-miR-17-10. It is known that functional miRNAs can have G:U pairing within the seed sequence (Didiano and Hobert 2006; Doench and Sharp 2004). To disrupt ovhv2-miR-17-10 binding, three amino acids (an arginine, an asparagine and a phenylalanine) were mutated. There are only two codons available for asparagine (AAC and AAT) and phenylalanine (TTC and TTT) and due to constraints of the genetic code mutation of these two amino acids results in changes in the base pairing of ovhv2-miR-17-10 with Ov2 from G:C to G:U. G:U pairing is allowed in seed sequences and can still result in functional knockdown of the target**.** Furthermore, RNAhybrid (Rehmsmeier et al. 2004) analysis using ovhv2-miR-17-10, allowing for G:U pairing and perfect complementarity between the Ov2 coding sequence and the miRNA seed sequence, identified two further possible and non-overlapping sites for ovhv2-miR-17-10 binding, in addition to the site tested. It is therefore possible that the reason Ov2 expression could not be restored, by mutation of the ovhv2-miR-17-10 binding site, is due both to the presence of functionally redundant ovhv2-miR-17-10 binding sites within the Ov2 mRNA or that ovhv2-miR-17-10 is still able to interact with the mutated Ov2 binding site via non-canonical G:U pairing.

Amongst cellular mRNAs, it has been shown that those that encode regulatory proteins, such as transcription factors, have a short half-life (Sharova et al. 2009; Yang et al. 2003). That Ov2 has a short half-life most likely explains the difficulties faced in detecting Ov2 transcript, despite being able to routinely detect protein and DNA in transfected cells. As a result it remains unclear whether ovhv2-miR-17-10 and ovhv2-miR-61-1 function to inhibit Ov2 expression via translational repression or transcriptional degradation. Previously, we have reported that ovhv2-miR-17-10 targets the OvHV-2 RTA transcript (Riaz et al. 2014). The observation that Ov2 is targeted by at least two viral-encoded miRNAs indicates that it has an important regulatory role in OvHV-2 biology. Using a reporter assay system, we have been able to demonstrate that Ov2 is capable of modulating RTA (See accompanying paper Dry et al. 2019). We propose that OvHV-2 utilises miRNAs ovhv2-miR-5 (Riaz et al. 2014), ovhv2-miR-17-10 and ovhv2-miR-61-1 to post-transcriptionally maintain latency, by restricting expression of RTA and Ov2, respectively, thereby increasing stringency to the process of reactivation by preventing precocious initiation of the productive lytic cycle.
